# Fighting fear in healthcare workers during the COVID-19 pandemic

**DOI:** 10.1017/ice.2020.315

**Published:** 2020-06-25

**Authors:** Kelly A. Cawcutt, Richard Starlin, Mark E. Rupp

**Affiliations:** Division of Infectious Diseases, University of Nebraska Medical Center, Omaha, Nebraska

“Fear is a reaction. Courage is a decision.”

Sir Winston Churchill

The current global coronavirus disease 2019 (COVID-19) pandemic is unprecedented and has stressed healthcare systems worldwide. Healthcare resources that are scarce include tests for severe acute respiratory coronavirus virus 2 (SARS-CoV-2), personal protective equipment (PPE), hospital equipment (ventilators), hospital capacity, and healthcare workers (HCWs), particularly those trained to care for the critically ill. Unfortunately, amid the pandemic and these shortages, anxiety and fear are rampant, fueled by real risk and amplified by the 24-hour news feed and social media.

The risk of acquiring infection is innate to health care; it always has been and, for the foreseeable future, will continue to be. Therefore, effective infection prevention practices are paramount to both ensuring safety and combatting fear. However, in the face of the COVID-19 pandemic, deviations in proven preventative measures and standard care are common. Variations in PPE use (eg, utilizing N95 respirators for minimal risk encounters) or deferring critical, life-saving procedures (ie, due to lack of confidence in validated diagnostic test performance or PPE efficacy) increase the overall risk to HCWs and patients alike. The reason these variations exist must be explored, and we postulate fear as a significant factor.

Among the many valid reasons for fear in this pandemic are fear of developing infection, fear of failing to provide adequate care for patients given limited resources, fear of carrying the virus home and infecting family and friends, fear of stigmatization, and many others. Fear is not novel to the COVID-19 pandemic; it has been well described in other infectious diseases epidemics such as HIV or SARS.^1^ Many of these fears are well founded considering reports of high rates of COVID-19 among frontline HCWs.^2^ Stigmatization of HCWs has already been described in association with COVID-19, as it was during the SARs epidemic in the early 2000s.^2^

Fear is powerful, and its influence in health care should not be underestimated. Fear is a negative emotion resulting in avoidance of specific stimuli based on perceived risk.^3^ In many situations, fear may be an appropriate reaction and can result in a decrease in engagement in at-risk behavior or greater adherence to mitigation strategies such as social distancing and handwashing.^3^ Unfortunately, fear has also been associated with maladaptive behaviors including overburdening of scarce resources (eg, demanding testing or medical attention when not needed), hoarding of precious supplies (eg, PPE), and failure to report for duty.^3^

Ho et al^1^ noted that fear in healthcare workers during SARS was significant. More than half of HCWs perceived low control over avoiding infection by complying with or maintaining infection prevention practices. High stress, heavy workload, and sudden changes in routine medical procedures during the SARS outbreak made it impossible for many HCWs to fully implement preventive practices, even though they understood their purpose and the potential risk of not following them.^1^ Fear resulting in overriding evidence-based practices carries increased risks for transmission of SARS-CoV-2 and other adverse events such as unnecessary avoidance of needed medical interventions. Interestingly, frontline HCWs caring for COVID-19 patients are reported to have less fear about becoming infected than HCWs in other units. This counterintuitive finding may be related to less direct education and communication with the lower risk group thereby failing to allay their concerns. Fear was also noted to be greater at the peak of the SARS epidemic among lower risk HCWs, which aligns with potential increased belief in self-perceived risk.^1^ This finding may correlate with inappropriate escalations of PPE or other unnecessary infection prevention practices and result in worsening of the critical PPE shortage. Furthermore, use of excessive or unfamiliar PPE may increase the risk of self-contamination and increase the risk of disease acquisition. If fear degrades confidence in infection prevention practices, including PPE use, even greater deviations from evidence-based practices may yet occur.

Strategies for addressing fear in such situations include targeted education to address fear, systemwide communication to avoid disparities in understanding, leveraging the call for altruism, emphasizing a sense of civic duty, encouraging colleagues to support each other, and encouraging those with a low fear threshold to seek available mental health support.^3-5^

Fear is commonplace with the COVID-19 pandemic. HCWs are not immune to anxiety and fear, and in fact, may suffer higher rates of fear than others. We must address the psychological impact of facing COVID-19 to further mitigate the spread of infection. We must remember that fear is a reaction and courage is the decision to trust tested infection prevention practices to provide the highest standard of care, in the safest environment that we can, for as long as we can. Choose courage.
